# Access matters: suprapubic-centered surgery as alternative to the transumbilical approach in laparoscopic right colectomy for colon cancer – a propensity score matched analysis

**DOI:** 10.1097/JS9.0000000000005087

**Published:** 2026-03-20

**Authors:** Hannes Hoi, Robert Uzel, Hannah Hofer, Lisa Schlosser, Helmut Weiss, Christof Mittermair

**Affiliations:** a Department of General and Visceral Surgery, St. John of God Hospital, Salzburg, Austria (Teaching Hospital of the Paracelsus Medical University Salzburg); b Department of Internal Medicine, St. John of God Hospital, Salzburg, Austria (Teaching Hospital of the Paracelsus Medical University Salzburg); c Department of Mathematics, University of Innsbruck, Innsbruck, Austria

**Keywords:** laparoscopic right colectomy, suprapubic approach, transumbilical approach

## Abstract

**Introduction:**

The Pfannenstiel incision has replaced the umbilicus as retrieval site in minimally invasive right colectomy. The aim of this study was to evaluate whether shifting the entire procedure towards a suprapubically centered access site provides additional advantages in terms of procedural safety, oncologic outcomes, recovery times and complication rates.

**Methods:**

A matched pair analysis was performed that compared 27 patients undergoing suprapubic laparoscopic right colectomy (study group) and 27 patients treated by transumbilical laparoscopic right colectomy (control group). Patients were matched based on age, gender and BMI. Total lymph node yield, conversion and complication rates, procedural duration, and length of stay were defined as primary outcome parameters.

**Results:**

Patients with a suprapubically centered surgery showed similar postoperative complication and conversion rates as compared to control patients. Suprapubic laparoscopic right colectomy was associated with the need for a higher number of additional working trocars (*P* < 0.05), showed a trend towards an increased total lymph node yield (*P* = 0.056), and significantly reduced the risk for postoperative incisional hernia development to 0% (*P* < 0.05).

**Conclusion:**

Suprapubic laparoscopic right colectomy for malignant colon cancer is safe and feasible, providing similar postoperative complication rates. This technique is favored due to a higher total lymph node harvest and a significant reduction of incisional hernia risk as compared to transumbilical laparoscopic right colectomy.

## Introduction

In times of a rapid development of surgical and targeted adjuvant pharmaceutical therapy for a variety of oncological diseases, laparoscopic right colectomy (LRC) has become a standardized and broadly implemented surgical procedure for the treatment of right-sided malignant colon cancer^[^1–3^]^. Regarding the surgical access site, a transumbilically centered port placement (TLRC) around a camera and working ports in the umbilical fossa is performed in the majority of cases. Favorable cosmetic results and low hernia rates have caused specimen retrieval to change from the umbilicus to a suprapubic Pfannenstiel incision[4]. Consequently, integration of the Pfannenstiel incision into the surgical strategy including working and camera port placement could be regarded as a conclusive consequence. However, a complete suprapubic approach for LRC (SLRC) has been described only in a small number of studies, providing potential advantages over TLRC^[^5–7^]^. These advantages are not limited to favorable outcomes in terms of a reduced incisional hernia risk but might offer improved vision along the axis of the superior mesenteric vessels[4]. This enables potentially more precise oncologic preparation[5]. Moreover, this approach reduces the risk of injury to surrounding anatomical structures due to better visibility and maneuverability as compared to the umbilical approach when robotic surgery is applied^[^8,9^]^.

HIGHLIGHTS

- Suprapubic laparoscopic right colectomy for malignant right-sided colon cancer is safe and feasible.
- This surgical strategy shows similar postoperative complication rates compared to transumbilical laparoscopic right colectomy.
- Suprapubic laparoscopic right colectomy significantly reduces the risk for incisional hernia development.

As comparative studies of TLRC and SLRC have not been conducted, this matched-pair analysis aims to clarify the impact that the surgical access site in laparoscopic right colectomy has on oncological outcome, patient safety in terms of conversion and complication rates and postoperative recovery.

## Methods

### Study design

This retrospective matched-pair analysis, including 54 patients undergoing laparoscopic right colectomy for colon carcinoma between January 2010 and December 2024 either via a suprapubic (27 patients) or a transumbilical access (27 patients), was conducted at a community-based hospital with a focus on minimally invasive surgery (MIS). Up to November 2022, the hospital’s standard treatment for right-sided colon cancer was LRC performed with a transumbilical approach. In December 2022, the surgical department changed its technical strategy for right-sided colon cancer in order to apply a standardized suprapubic approach for LRC in all patients. Procedural and demographic data of all consecutive patients undergoing SLRC were collected and matched with 27 corresponding TLRC procedures (performed between January 2010 and November 2022).

### Data acquisition/measurements

Data were collected with the hospital’s internal software system “Patidok 2.0” and exported to a Microsoft Excel database (Microsoft Excel, Microsoft Corporation, Redmond, Washington (WA), USA). Data analysis comprised patient characteristics (age, gender, BMI, American Association of Anaesthesiologists = ASA classification, types of previous abdominal surgeries), procedural data (date of surgery, procedural duration in minutes, expertise level of operating surgeon, conversion rate from MIS to open surgery (OS), number of trocars, type of ileotransverse anastomosis (intracorporeally stapled versus extracorporeally hand sewn, intraoperative complications) and outcome parameters (lymph node yield (total number and number of positive lymph nodes), Union for International Cancer Control = UICC classification, length of stay (LOS), complication rates, postoperative 30-day readmission rate to hospital).

The work has been reported in line with the STROCSS criteria[10].

### Inclusion criteria

Patients undergoing laparoscopic right colectomy for malignant disease.

Patients older than 18 years.

### Exclusion criteria

- Patients undergoing laparoscopic right colectomy for benign disease.
- Emergency surgeries (laparoscopic right colectomy with active tumor bleeding, perforation, or acute bowel obstruction).

### Surgical technique

SLRC was performed using a suprapubic port placement, consisting of a single-port system placed in the midline two to three finger widths above the pubic symphysis (for camera and both right and left instruments of the surgeon) and optionally one or two additional trocars (for the assisting surgeon or anastomotic suturing). For TLRC, a single-port system was placed directly at the level of the umbilicus (for camera and both right and left surgical instruments), being optionally accompanied by one or two additional trocars (for the assisting surgeon or anastomotic suturing). Both procedures followed standard oncological principles, including complete mesocolic excision (CME) and D2 lymphadenectomy. The decision on the order of preparation steps (starting from bottom-to-up, from supramesocolic, from the lateral or medial side) was left to the surgeon’s preference. Concerning reconstruction of the intestinal passage, intracorporeally stapled anastomosis using one 60-mm cartridge with suturing of the remaining enterotomy or extracorporeal hand-sewn anastomosis was performed in both groups.

### Primary outcome parameters

- Lymph node yield: Measured as the mean number of lymph nodes measured by means of histopathological workup.
- Procedural duration: Time from skin incision to skin closure.
- Conversion to OS: number and percentage of conversions from laparoscopic to open surgery
- Postoperative complication rates: including anastomotic leakage, bleeding, wound infection and burst abdomen.
- Length of hospital stay: days from the day of surgery to patient discharge.

### Secondary outcome parameters

- One-year tumor recurrence rates (locoregional recurrence and metastatic disease)
- Incisional hernia rates (hernia detection was performed with the help of computed tomography that was carried out as part of oncologic follow-up examination: 12, 24, and 60 months after surgery)

### Analysis:

Statistical analysis was performed by a mathematician not involved in patient assessment using R, version 4.0.5. All statistical calculations were two-sided, and a significance level of 5% was applied.

Matching was performed using nearest-neighbor matching based on the *propensity score*, estimated via logistic regression using the MatchIt package in R, with the aim of achieving balance between groups with respect to age, sex, and body mass index (BMI). After matching, the balance of matching variables between groups was assessed using paired statistical tests (McNemar’s chi-squared test for categorical variables and the Wilcoxon signed-rank test for continuous variables), confirming the absence of statistically significant differences between the comparison groups on the matching variables.

The same paired statistical tests were subsequently applied for group comparisons of the outcomes of interest. Continuous data are presented as median (25th–75th percentile), and categorical variables as frequencies and percentages. Effect sizes and precision are reported as estimated median differences between groups for continuous outcomes and odds ratios (ORs) for binary outcomes, each with corresponding 95% confidence intervals (CIs).

Sample size estimation was performed based on the outcome parameter “incisional hernia,” as no profound data on any other outcome parameter is available. According to data reported in the literature, the expected incidence was 0% in the suprapubic group and 29% in the umbilical group (Lee *et al*, 2012)[4]. Assuming a two-sided alpha level of 5% and a target power of 80%, these expected proportions yielded a required sample size of 22 participants per group for a Fisher’s Exact Test. To account for potential dropouts during the study and possible uncertainties in the sample size calculation (e.g., regarding the choice of statistical test), the final sample size was set at 27 participants per group.

A post-hoc power analysis was performed for the parameter “lymph node yield” using a paired t-test. Statistical power of the Wilcoxon Signed Rank Test used in the preceding analysis revealed a value of 0.96.

## Results

A total of 54 patients were included in the analysis, with 27 patients in the SLRC and 27 in the TLRC group.

As age (*P* = 0.2920), BMI (*P* = 0.1331) and gender (11 males and 16 females in each group) served as matching criteria between SLRC and TLRC, no significant differences were observed between the groups. Regarding other patient characteristics, no significant differences between the two groups were found in terms of demographics or comorbidities, including ASA scores and number and type (minor and major) of previous abdominal surgeries. Results are shown in Table 1.

**Table 1:** Comparison of patient characteristics in SLRC and TLRC.

	Total (*n* = 54)^a^	SLRC (*n* = 27)	TLRC (*n* = 27)	Estimate with 95% CI^b^	*P*-Value (paired)^c^
ASA Score	2 (2–3)	3 (2–3)	2 (2–3)	0 (0 to 1)	0.1111
Age at time of surgery in years	78 (74–83.75)	78 (74–83)	78 (74–84.5)	0 (−4 to 4)	0.2920
BMI	25.3 (23.4–28)	25.1 (23.5–28.55)	25.7 (23–27.85)	0.4 (−1.9 to 2.4)	0.1331
Sex (female)	32/54 (59.3%)	16/27 (59.3%)	16/27 (59.3%)	1 (0.29 to 3.39)	1
No history of previous abdominal surgery	15/54 (27.8%)	7/27 (25.9%)	8/27 (29.6%)	1.2 (0.31 to 4.74)	1
Minor previous surgery (appendectomy, cholecystectomy, hysterectomy, adnexectomy, fundoplication, caesarean section)	31/54 (57.4%)	15/27 (55.6%)	16/27 (59.3%)	1.16 (0.35 to 3.92)	1
Major previous surgery (bowel resection; bowel obstruction; liver, kidney, and prostate resection; vascular bypass)	8/54 (14.8%)	5/27 (18.5%)	3/27 (11.1%)	0.56 (0.08 to 3.25)	0.7237
Neoadjuvant chemotherapy	1/54 (1.9%)	1/27 (3.7%)	0/27 (0%)	Inf (0.03 to Inf)	1

^a^ Binary data are presented as no./total no. (%), continuous data as median (25th to 75th percentile).

^b^ Odds ratios for binary variables and estimated median difference for continuous variables.

^c^ Assessed by means of McNemar’s chi-squared test for categorical variables and Wilcoxon Signed Rank Test for continuous variables.

Concerning procedural data, operation time did not differ significantly between SLRC and TLRC (143 minutes versus 133 minutes, respectively; *P* = 0.6394). Ileotransverse anastomosis was performed laparoscopically in all patients in the SLRC group, whereas nine (33.3%) patients in the TLRC group underwent an extracorporeal hand-sewn anastomosis (*P* < 0.05). In addition to the single-port system, accessory trocars were used in 14 (51.9%) patients in the SLRC cohort and in five (18.5%) patients in the TLRC group (*P* < 0.05). There were no intraoperative conversions to an open procedure in either the SLRC or the TLRC group. Results are shown in Table 2.

**Table 2:** Comparison of procedural data in SLRC and TLRC.

	Total (*n* = 54)^a^	SLRC (*n* = 27)	TLRC (*n* = 27)	Estimate with 95% CI^b^	*P*-value (paired)^c^
Procedural duration (minutes)	138.5 (115.75–179)	143 (119–173.5)	133 (108–179.5)	9 (−17 to 31)	0.6394
Experience level of surgeon = senior specialist	42/54 (77.8%)	21/27 (77.8%)	21/27 (77.8%)	1 (0.23 to 4.43)	1
Number of additional trocars used	0 (0–1)	1 (0–1)	0 (0–0)	0 (0 to 1)	0.1078
Use of additional trocars	19/54 (35.2%)	14/27 (51.9%)	5/27 (18.5%)	0.22 (0.05 to 0.83)	0.0265
Number of intracorporeal anastomoses	45/54 (83.3%)	27/27 (100%)	18/27 (66.7%)	Inf (2.51 to Inf)	0.0077
Number of intraoperative complications	2/54 (3.7%)	1/27 (3.7%)	1/27 (3.7%)	1 (0.01 to 81.43)	1 (all equal)

^a^ Binary data are presented as no./total no. (%), continuous data as median (25th to 75th percentile).

^b^ Odds ratios for binary variables and estimated median difference for continuous variables.

^c^ Assessed by means of McNemar’s chi-squared test for categorical variables and Wilcoxon Signed Rank Test for continuous variables.

Histopathologic examinations of specimens revealed an average harvest of 26 lymph nodes in the SLRC and 22 nodes in the TLRC group (*P* = 0.056), with a median positivity of 0 (0–3) nodes in the SLRC group and 0 (0–0.5) nodes in the TLRC cohort (*P* = 0.1161).

Postoperative complication rates (complications ≥ Dindo 3b) were 22.2% (6/27 patients) in the SLRC and 25.9% (7/27 patients) in the TLRC group. Thereof, anastomotic leakage occurred in 11.1% (3/27 patients) in the SLRC and the TLRC group each. Postoperative bleeding was observed in 11.1% (3/27 patients) undergoing SLRC and 3.7% (1/27 patients) treated by TLRC (*P* = 0.6171). The 30-day readmission rate was 3.7% in the SLRC and the TLRC group and did not include hernia-related admissions. Results are shown in Table 3.

**Table 3:** Comparison of postoperative complication rates and outcome in SLRC and TLRC.

	Total (*n* = 54)^a^	SLRC (*n* = 27)	TLRC (*n* = 27)	Estimate with 95% CI^b^	*P*-Value (paired)^c^
Number of complications	15/54 (27.8%)	9/27 (33.3%)	6/27 (22.2%)	1.73 (0.45 to 7.17)	0.5465
Complications Clavien-Dindo < 3b	41/54 (75.9%)	21/27 (77.8%)	20/27 (74.1%)	0.82 (0.19 to 3.42)	1
Complications Clavien-Dindo ≥ 3b	13/54 (24.1%)	6/27 (22.2%)	7/27 (25.9%)	1.22 (0.29 to 5.24)	1
Incisional hernia rates	6/54 (11.1%)	0/27 (0%)	6/27 (22.2%)	0 (0 to 0.76)	0.0412
Readmission to hospital 30 days after surgery	2/54 (3.7%)	1/27 (3.7%)	1/27 (3.7%)	1 (0.01 to 81.43)	1
Postoperative bleeding	4/54 (7.4%)	3/27 (11.1%)	1/27 (3.7%)	3.18 (0.24 to 176.87)	0.6171
Anastomotic leakage	6/54 (11.1%)	3/27 (11.1%)	3/27 (11.1%)	1 (0.12 to 8.24)	1
Length of stay (days)	9 (7–13)	8 (7–12.5)	10 (8–13)	−2 (−4 to 0)	0.3628

^a^ Binary data are presented as no./total no. (%), continuous data as median (25th to 75th percentile).

^b^ Odds ratios for binary variables and estimated median difference for continuous variables.

^c^ Assessed by means of McNemar’s chi-squared test for categorical variables and Wilcoxon Signed Rank Test for continuous variables.

Regarding complication management, all anastomotic leaks were managed with anastomotic resection and renewal of the ileocolonic anastomosis. Anastomotic bleeding in the SLRC group was managed with endoscopic clip application in two patients. One patient in each group. underwent redo surgery for retroperitoneal bleeding control. Another patient in the TLRC group suffered from rectal perforation due to transabdominal drainage injury, which was dealt with simple suturing. One patient in the TLRC group underwent laparoscopic exploration without pathologic finding.

Incisional hernia rates in the TLRC group were 18.5% (5/27) and 22.2% (6/27 cases) one and five years after surgery, respectively, whereas no hernias were found in the SLRC group (*P* < 0.05). Absolute risk reduction (ARR) yielded 0.22 and the number needed to treat (NNT) is five.

Median length of stay was 8 days (7–12.5) in the SLRC group versus ten days (8–13) in the TLRC cohort (*P* = 0.36). Median UICC (Union for International Cancer Control) stage did not differ between SLRC and TLRC (UICC 2 in both groups). Median follow-up was 5 years in the TLRC group and 1 year in the SLRC group. Only systemic recurrence (2 out of 27 patients; 7.4% in both the TLRC and the SLRC group) occurred and no locoregional recurrence could be found after 1 year (Table 4).

**Table 4:** Group comparison of oncologic outcomes in SLRC and TLRC.

	Total (*n* = 54)^a^	SLRC (*n* = 27)	TLRC (*n* = 27)	Estimate with 95% CI^b^	*P*-Value (paired)^c^
Number of positive lymph nodes	0 (0–1)	0 (0–3)	0 (0–0.5)	0 (0 to 1)	0.1161
Total number of lymph nodes in specimen	23 (17.25–29.75)	26 (21.5–30)	22 (16–27.5)	5 (−1 to 9)	0.0559
UICC < 2	16/54 (29.6%)	10/27 (37%)	6/27 (22.2%)	0.49 (0.12 to 1.86)	0.4227
UICC < 3	36/54 (66.7%)	16/27 (59.3%)	20/27 (74.1%)	1.94 (0.54 to 7.4)	0.4227
UICC < 3a	36/54 (66.7%)	16/27 (59.3%)	20/27 (74.1%)	1.94 (0.54 to 7.4)	0.4227
UICC < 3b	40/54 (74.1%)	18/27 (66.7%)	22/27 (81.5%)	2.17 (0.54 to 9.81)	0.3865
UICC < 3c	47/54 (87%)	23/27 (85.2%)	24/27 (88.9%)	1.38 (0.21 to 10.49)	1
UICC < 4	51/54 (94.4%)	26/27 (96.3%)	25/27 (92.6%)	0.49 (0.01 to 9.91)	1
Recurrent disease	4/54 (7.4%)	2/27 (7.4%)	2/27 (7.4%)	1 (0.07 to 14.8)	1

^a^ Binary data are presented as no./total no. (%), continuous data as median (25th to 75th percentile).

^b^ Odds ratios for binary variables and estimated median difference for continuous variables.

^c^ Assessed by means of McNemar’s chi-squared test for categorical variables and Wilcoxon Signed Rank Test for continuous variables.

Cost analysis: Regarding surgical equipment, the same single port systems and re-usable trocars were used in SLRC and TLRC, thus reflecting no procedural differences in material costs.

## Discussion

The results of this matched-pair analysis reveal that suprapubic laparoscopic right colectomy (SLRC) for malignant right-sided colon cancer is safe and feasible, offering an alternative surgical approach with similar postoperative complication rates, a superior view along the mesenteric axis and a trend towards favorable oncologic outcomes in terms of total lymph node harvest, as compared to transumbilical laparoscopic right colectomy (TLRC). Moreover, the risk for postoperative incisional hernia development was able to be significantly reduced from 22.2% in TLRC to 0% in SLRC.

Regarding a higher likelihood for incisional hernia development associated with TLRC, similar findings were made by Greemland *et al* in an investigation of 370 patients undergoing right colectomy[11]. More precisely, the authors revealed a significant decrease in the risk for postoperative incisional hernia development when the specimen was extracted off-midline. In another retrospective analysis of incisional hernia rates 1 year after laparoscopic colorectal resections with specimen retrieval either via midline or suprapubic incision in 168 patients, a significantly higher risk for hernia development was seen when the bowel was delivered via the umbilicus (13.3% versus 0%; *P* = 0.001)[12]. Therefore, SLRC may combine these favorable aspects of hernia prevention with regards to specimen extraction with reduced trauma to the abdominal wall, when (peri-)umbilical trocar placement is no longer necessary, resulting not only in better cosmetic outcomes but also potentially reducing postoperative pain[13]. Even if the risk for postoperative incisional hernia development in colorectal surgery is known to be highest in the first year after primary surgery[14], a shorter median follow-up period of only 1 year in the SLRC group in our analysis should be mentioned. However, it should be mentioned that all but one incisional hernias in the TLRC group occurred during the first year after surgery.

**Figure 1.**
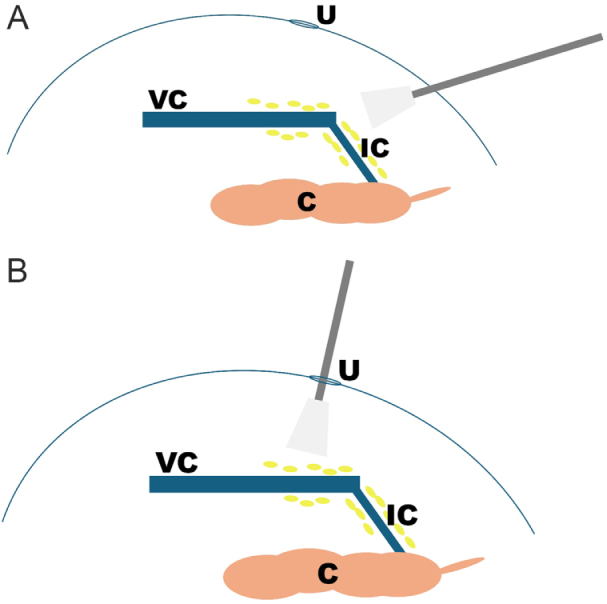
(A and B) Surgeon’s view on the mesenteric axis during SLRC and TLRC. *1a: Surgeon´s view on the mesenteric axis during SLRC. C, colon; IC, ileocolic vein; U, umbilicus; VC, vena cava.1b: Surgeon´s view on the mesenteric axis during TLRC. C, colon; IC, ileocolic vein; U, umbilicus; VC, vena cava.*

Another advantage of SLRC is a better overview along the axis of the mesenteric vessels (Fig. 1a and b). Lymphadenectomy, central vessel ligation and the optimal vision of the axis of the superior mesenteric vein and artery are in the line of sight with the suprapubic access, thus facilitating these essential preparation steps[5]. Moreover, the surgeon´s visual perspective from the suprapubic area offers a favorable distance between camera, working ports and the target organ (right colon), that allows for the maintenance of a good overview throughout the entire procedure. Bearing in mind the open book model, consisting of four symbolic pages representing the corresponding dissection planes (retroperitoneal, ileocolic, transverse mesocolic, and mesogastric) with their vascular relations[15], the turning of pages may be more intuitive with SLRC. On the other hand, a short distance and an impractical angulation for tissue preparation between the umbilical fossa and the superior mesenteric vessels may hamper preparation techniques in TLRC. This is underlined by a trend towards a higher total lymph node harvest in patients treated by means of SLRC and may reflect a simplification of technical aspects of complete mesocolic excision (CME), achieved with this access strategy. As data are lacking in the current literature this novel finding will be of utmost importance for the oncologic outcome if this is confirmed in larger studies.

Our standard for anastomosis in the right colon is the intracorporeal technique. This has been shown to have advantages over extracorporeal anastomosis^[^16–18^]^. However, it has to be emphasized that the TLRC approach provides advantages in terms of surgeon familiarity and easier initial trocar placement, particularly for surgeons without experience in the suprapubic technique. The relative proximity to the transverse colon may simplify extracorporeal anastomotic techniques in some patients. On the other hand, the short distance to and the steep angle between the anastomosis and the access route may hamper the reconstruction in others. It can be assumed that the use of additional trocars may not further improve this technical obstacle of angulation. This is underlined by a scarce use of additional trocars and a significantly higher rate of extracorporeal anastomoses in the TLRC group as compared to the SLRC cohort. On the other hand, it can be assumed that the potentially long distance between the suprapubic working ports and the ileotransverse anastomosis may complicate tissue handling and exposure. This is reflected by a significantly higher use of additional trocars in the SLRC group. Due to the long distance between the suprapubic incision and the transverse colon, extracorporeal anastomosis is hardly possible in the SLRC group. However, this technical problem may be solved with the aid of stable robotic platforms, enabling the surgeon to overcome the aforementioned difficult situations. In particular, robotic single-port systems, placed in the suprapubic area, may combine the advantages of cosmetic and technical issues as well as a reduced risk for incisional hernia development.

Although not significant, a longer procedural duration (10 minutes longer) in the SLRC group may be of multifactorial origin. Firstly, the departmental surgical strategy to opt for a suprapubic access in all patients undergoing LRC is relatively new (starting in December 2022), hence reflecting the very first results for this special technique performed at the hospital. Secondly, the fact that the distance between the suprapubic working ports and the transverse colon in SLRC is longer as compared to the distance from the umbilical fossa may influence the difficulty of intracorporeal ileotransverse anastomotic reconstruction. Thirdly, in patients presenting with anatomical variations where the cecum is found deep in the small pelvis or those with a significant amount of adhesions in this dissection area (patients with prior appendectomy or gynecologic surgery), the distance to the suprapubic working and camera ports is rather short. This results in more difficult adhesiolysis and especially in mobilization of the ileocecal region in these patients.

Lastly, differences in the access route should under no circumstances impair complication rates or oncologic outcome as tissue handling and preparatory steps follow the same rules for LRC in both SLRC and TLRC.

### Limitations

As this study is one of the first to compare SLRC and TLRC no profound data is available to compare these findings. Concerning the retrospective, single-center character of this study, larger multicenter randomized-controlled trials (RCTs) are needed to confirm the findings of this matched-pair analysis. The relatively small patient cohort and short follow-up period in the SLRC group have to be regarded as limitations, concerning both the oncologic recurrence and incisional hernia risk. These should include technical standardization of procedural steps like anastomotic reconstruction (intra- versus extracorporeal and stapled versus hand-sewn anastomosis).

Furthermore, the learning curve for SLRC was not evaluated in this study protocol.

## Conclusion

Right colectomy via the suprapubic approach (SLRC) is a safe and feasible procedure. Compared to the transumbilical route the advantage of a decreased risk for incisional hernia is paralleled by a trend to a more sufficient lymph node harvest that has the potential to improve the oncologic outcome.

## Data Availability

The datasets generated and analyzed in the current study are not publicly available due to hospital policy but are available from the corresponding author on reasonable request.

## Research registration unique identifying number (UIN)

Retrospectively registered with the German Clinical Trials Register: DRKS 000036743.
